# Supplementary material for: Potential Impacts of Prunus domestica Based Natural Gum on Physicochemical Properties of Polyaniline for Corrosion Inhibition of Mild and Stainless Steel
Source: Polymers (Basel). 2022 Jul 30;14(15):3116. doi: 10.3390/polym14153116 (PMC9370546; doi:10.3390/polym14153116)
Supplement: Supplementary file 1 [file polymers-14-03116-s001.zip › polymers-1783579-supplementary.pdf]

Supplementary information

Potential Impacts of *Prunus Domestica* Based Natural Gum on Physicochemical Properties of Polyaniline for Corrosion Inhibition of Mild and Stainless Steel

Muhammad Kamran[1], Anwar ul Haq Ali Shah[1], Gul Rahman[1], Salma Bilal[2*]

[1]Institute of Chemical Sciences, University of Peshawar, 25120 Peshawar, Pakistan
[2]National Centre of Excellence in Physical Chemistry, University of Peshawar, 25120 Peshawar, Pakistan
* Corresponding author
Salma Bilal
E.mail : salmabilal@uop.edu.pk

3.1. Optimization

PDG-g-PANI composites were optimized by varying one reactant and keeping all other reactants constant. The resultant product was characterized by TAFEL plots. The results of the optimization are given below.

3.1.1. Effect of Monomer (Aniline)

Aniline monomer amount were optimized by varying the concentration of aniline while keeping the concentration of all other reactants e.g PDG, DBSA oxidant and solvent constant. The optimization of aniline was started from 0.1 mL aniline but the product obtained at 0.1, 0.2 and 0.3 mL aniline has comparatively low solubility and very poor adhesion property and hence its corrosion protection ability was also very low. This can be attributed to formation of long chain polyaniline fabricated with PDG composites. But when the amount of aniline reached to 0.4 mL, a different type of product was formed in the form of grain as shown in the Figure 1, which had completely different properties than the product as mentioned above. At this

concentration of aniline small polymer chain having interaction with galactoarabinose components of PDG occurs having enhanced solubility and good corrosion protection ability on mild as well as stainless steel. Then the concentration of the aniline was further varied and their corrosion protection ability was tested and the composites were coded with special symbols. After changing the concentration of aniline beyond 0.4 mL a similar product having good solubility but low corrosion protection was obtained.

Table S1. Effect of amount of monomer (aniline) on corrosion rate of PDG-g-PANI

Sample name	Aniline (mL)	DBSA (mL)	PDG (gm)	Diesel mL	Water (mL)	Oxidant (gm)	Corrosion rate (m/year)
0.4 A	0.4	1	0.1	30	30	1	0.350
0.5 A	0.5	1	0.1	30	30	1	0.786
0.6 A	0.6	1	0.1	30	30	1	1.01

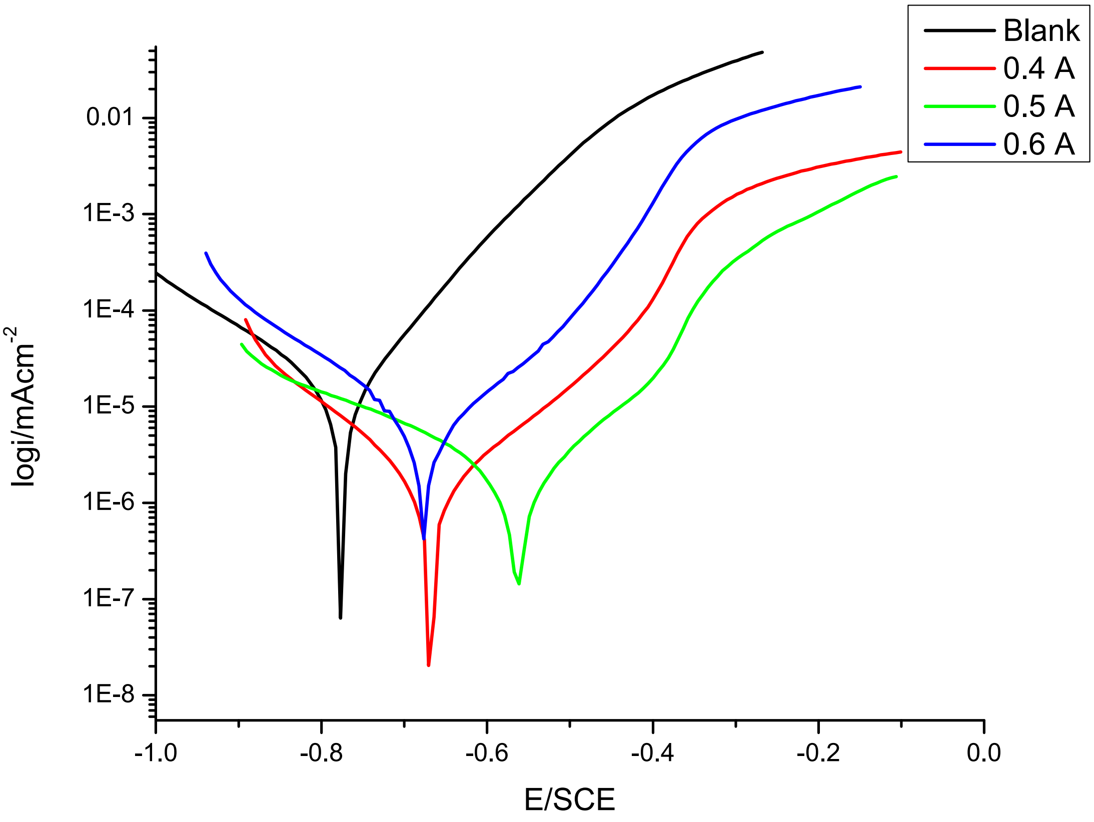

Figure S1. Tafel plot of aniline optimized samples.

3.1.2. Effect of Oxidant (BPO)

After optimizing aniline concentration benzoyl peroxide (oxidant) concentration were optimized. Amount of aniline along with other reactants were kept constant while amount of oxidant was varied from 0.2 gm to 1.2 gm. At low concentration of oxidant the polymerization was slow and small polymer chain (oligomers) was obtained. However with increasing amount of oxidant the polymerization process enhanced and interaction of PDG with polymer chain occurs resulting in composite formation. The interaction of PDG with polyaniline is hydrogen bonding where –OH groups of galactoarabinose interact with –NH groups of aniline in the

polymer chain. At 1 gm of benzoyl peroxide the product formed had excellent corrosion protection ability on steel.

Table S2. Effect of amount of oxidant (BPO) on corrosion rate of PDG-g-PANI.

Sample name	Aniline (mL)	DBSA (mL)	PDG (gm)	Diesel mL	Water (mL)	Oxidant (gm)	Corrosion rate (m/year)
0.2 B	0.4	1	0.1	30	30	0.2	4.24
0.4 B	0.4	1	0.1	30	30	0.4	1.74
0.6 B	0.4	1	0.1	30	30	0.6	2.29
0.8 B	0.4	1	0.1	30	30	0.8	0.556
1.0 B	0.4	1	0.1	30	30	1.0	0.508
1.2 B	0.4	1	0.1	30	30	1 .2	3.25

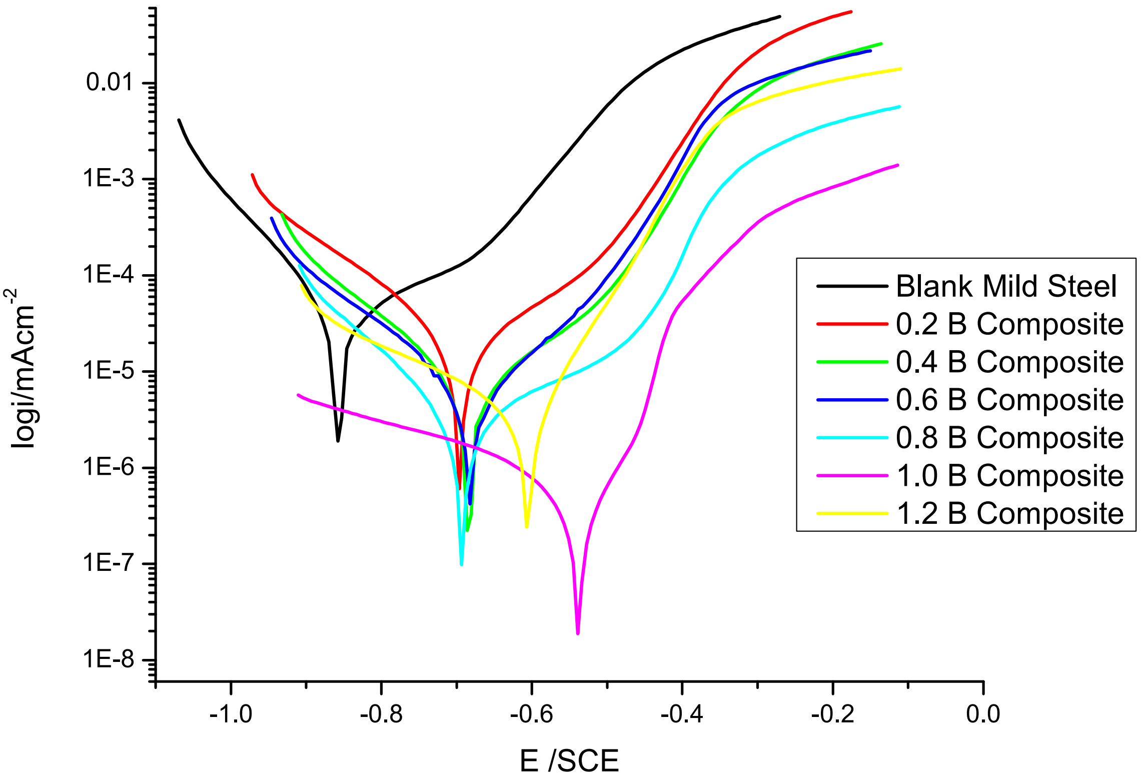

Figure S2. Tafel plot of oxidant optimized samples

3.1.3. Effect of PDG

Effect of amount PDG on the properties of resultant composite was also analyzed. In this study the optimized values of aniline, BPO and DBSA were kept constant while amount of PDG was varied from 0.05 gm to 0.25 gm. The product having best properties was selected on the basis of corrosion protection ability and the results are shown in the Table 3. At low concentration of gum components of PDG make interactions with –NH at certain position, leaving some positions unblocked, while at higher concentration of PDG, –OH groups of galactoarabinose make interaction with almost all sites blocking –NH positions throughout the chain. So at low concentration of gum the composite shows good protection behavior against corrosion while at higher concentration the protection ability slightly decreases. When some binding moieties of polyaniline chain (-NH) are vacant it make strong interaction with "Fe" atoms in mild steel, and show protection ability by redox mechanism while PDG in composite can make interaction with "Fe" atoms and show corrosion protection ability by making a strong hydrophobic insulating layer.

So on the basis of corrosion protection ability 0.1 G composite were selected as optimized sample.

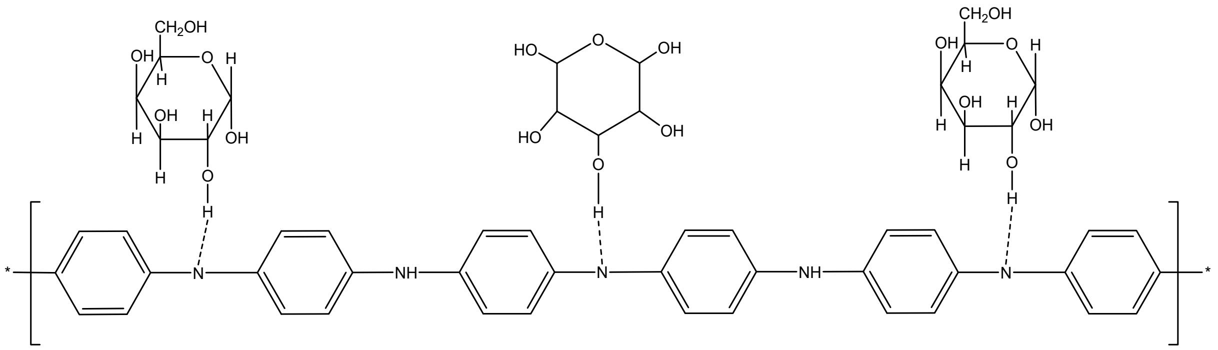

Table S3. Effect of amount of PDG on corrosion rate of PDG-g-PANI.

Sample name	Aniline (mL)	DBSA (mL)	PDG (gm)	Diesel (mL)	Water (mL)	Oxidant (gm)	Corrosion rate (m/year)
0.05 G	0.4	1	0.05	30	30	1	0.482
0.1 G	0.4	1	0.10	30	30	1	0.315
0.15 G	0.4	1	0.15	30	30	1	0.973
0.2 G	0.4	1	0.20	30	30	1	2.54

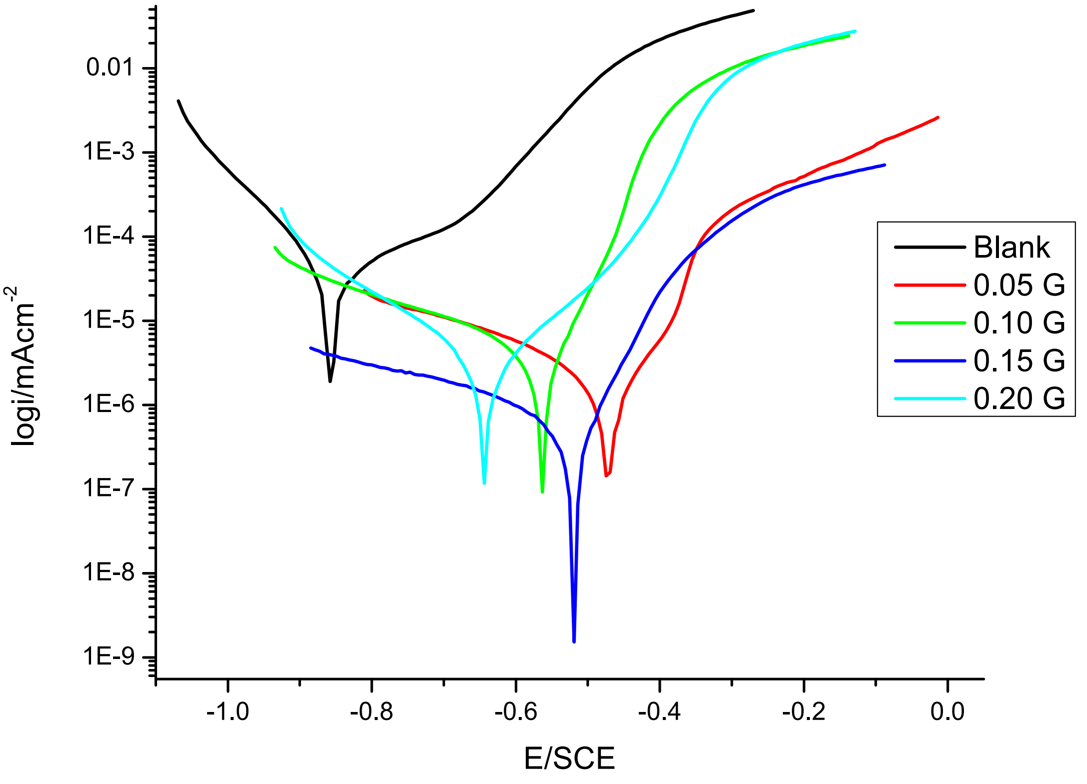

Figure S3. Tafel plots of different gum optimized samples

3.1.4. DBSA Optimization

After optimizing aniline, BPO and PDG amount of surfactant, dopant (DBSA) were also optimized. DBSA both act as surfactant and dopant. DBSA enhances the solubility of composites

in common organic solvents and hence increases its processesability. At 1 mL DBSA the composite show good corrosion protection ability.

Table S4. Effect of amount of surfactant (DBSA) on corrosion rate of PDG-g-PANI

Sample name	Aniline (mL)	DBSA (mL)	PDG (gm)	Diesel (mL)	Water (mL)	Oxidant (gm)	Corrosion rate (m/year)
0.3 D	0.4	0.3	1	30	30	1	2.73
0.6 D	0.4	0.6	1	30	30	1	1.13
1.0 D	0.4	1.0	1	30	30	1	0.742
1.2 D	0.4	1.2	1	30	30	1	0.890
1.5 D	0.4	1.5	1	30	30	1	0.813

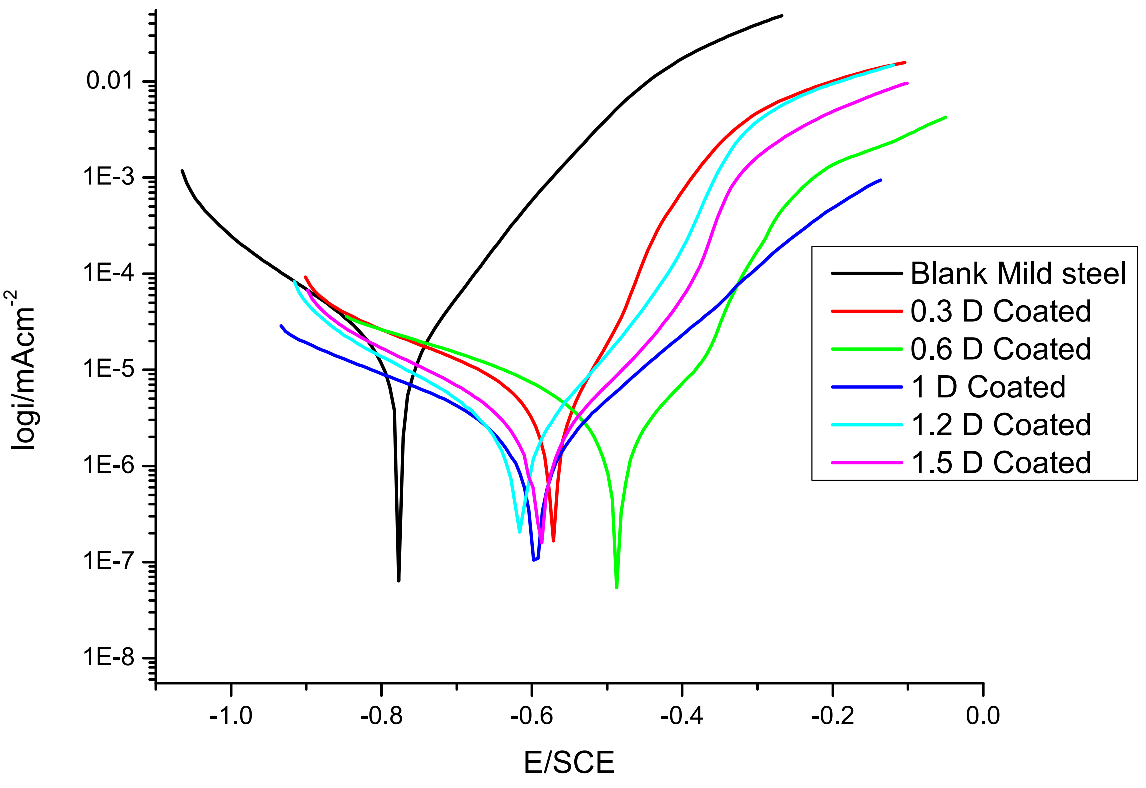

Figure S4. Tafel plots of DBSA optimized samples
